# Chain mediating effect of somatic pain and sleep duration between chronic disease count and depressive symptoms in older adults

**DOI:** 10.3389/fpsyt.2026.1821807

**Published:** 2026-07-22

**Authors:** Lan Ma

**Affiliations:** Department of Pain, The First People’s Hospital of Zunyi, Guizhou, China

**Keywords:** chain mediating effect, chronic diseases, depressive symptoms, older adults, sleep duration, somatic pain

## Abstract

**Background:**

Population aging has intensified concerns regarding mental health in older adults. Multimorbidity is highly prevalent in late life and is strongly associated with both somatic pain and depressive symptoms. Somatic pain and abnormal sleep duration are common in older adults and may mediate the relationship between chronic disease burden and depression. However, the potential chain mediating roles of somatic pain and sleep duration between chronic disease count and depressive symptoms remain unclear.

**Aim:**

To examine whether somatic pain and sleep duration independently and sequentially mediate the association between chronic disease count and depressive symptoms in older adults.

**Methods:**

A total of 213 older adult patients who visited the First People’s Hospital of Zunyi from March 2020 to March 2024 were selected as the research subjects. Data were collected using a general information questionnaire, the Chronic Disease Checklist, the Short-Form McGill Pain Questionnaire (SF-MPQ), and the 10-item Short-Form Center for Epidemiological Studies Depression Scale (CES-D-10). SPSS 26.0 and PROCESS 4.1 macro program were used for correlation analysis, regression analysis, and bootstrap mediation effect test.

**Results:**

The depression detection rate was 40.38%. Chronic disease count, sleep duration, somatic pain, and depressive symptoms were significantly correlated (all P < 0.01). After covariate adjustment, chronic disease count had a significant direct effect on depressive symptoms (β=0.164, 95% CI: 0.056–0.273). The independent mediating effects of somatic pain (effect=0.122, 95% CI: 0.057–0.187) and sleep duration (effect=0.054, 95% CI: 0.021–0.096), as well as the chain mediating effect of “somatic pain → sleep duration” (effect=0.018, 95% CI: 0.006–0.037), were all significant. The total indirect effect accounted for 54.17% of the total effect.

**Conclusion:**

Somatic pain and sleep duration independently and sequentially mediate the association between chronic disease count and depressive symptoms in older adults. Integrated strategies targeting multimorbidity control, pain management, and sleep regulation may help reduce depression risk. However, the chain-mediated effect was small in magnitude, suggesting that direct interventions targeting pain and sleep may offer more clinically meaningful benefits than long-chain approaches. Given the cross-sectional design, causal directions cannot be inferred.

## 1 Introduction

Chronic diseases are a major threat to the life and health of older adults. Multimorbidity—the coexistence of two or more chronic conditions—afflicts the majority of older adults and markedly impairs physical and mental function, quality of life, and survival. Against the backdrop of rapid population ageing, the number of older adults living with multimorbidity is steadily increasing (1). The high prevalence of chronic illness exposes older people to persistent somatic pain; more than two-thirds report persistent pain complaints (2). Somatic pain is an unpleasant bodily sensation linked to tissue damage, and chronic-disease complications can intensify pain in multiple body regions (3). Individuals suffering from pain frequently experience sleep disturbances—shortened sleep duration, reduced sleep quality, and insomnia (4, 5). Evidence indicates that the likelihood of sleep problems among people in pain is up to 70% higher than among their pain-free counterparts (6). Prolonged pain not only curtails sleep but may also contribute to systemic inflammation and hypothalamic-pituitary-adrenal (HPA) axis hyperactivity (7, 8), and disturbs serotonin and norepinephrine metabolism, key regulators of sleep and mood (9, 10). These physiological changes jointly disrupt the sleep-wake cycle and further induce negative emotional states (11).

Globally, more than 50% of patients with chronic pain exhibit depressive symptoms (12). Depression in later life undermines appetite, lowers mood, fuels anxiety, and increases suicide risk (13). In primary care, chronic pain is one of the most demanding long-term conditions in terms of consultation rates and costs (14). To date, most studies have examined pairwise links among chronic disease, pain, and depression, leaving the interrelationship among all three underexplored. In particular, whether somatic pain and sleep duration mediate the association between chronic disease burden and depressive symptoms remains insufficiently investigated.

**Figure 1:**
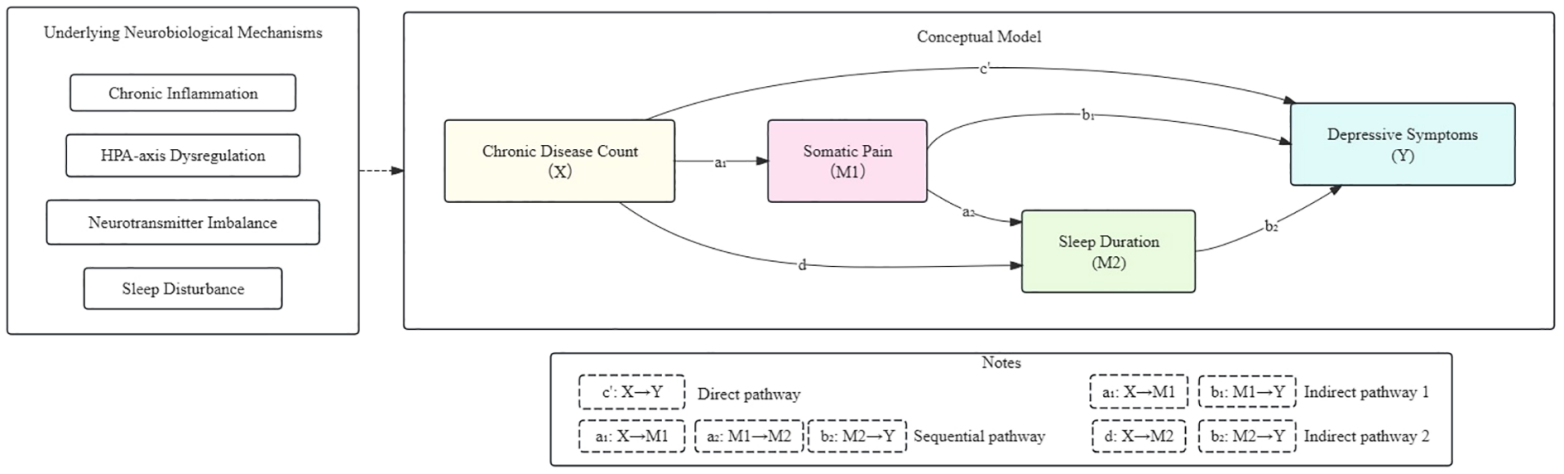
Schematic of the hypothesized chain association model among chronic disease count, somatic pain, sleep duration, and depressive symptoms. All paths indicate statistical associations, not causality.

Based on pathophysiological evidence, we propose a chain mediation model: chronic disease count (X) is associated with greater somatic pain (M1), which is associated with shorter sleep duration (M2), and both M1 and M2 are associated with higher depressive symptoms (Y). This temporal sequence is clinically plausible because chronic diseases are long-standing conditions that predispose to pain, which in turn disrupts sleep and mood (21, 22). We hypothesize that: (1) chronic disease count is directly associated with depressive symptoms; (2) somatic pain independently mediates this association; (3) sleep duration independently mediates this association; and (4) somatic pain and sleep duration form a sequential (chain) mediating pathway (**Figure 1**). By testing this model, we seek to elucidate the intrinsic mechanisms underlying the clustering of health problems in later life and provide a theoretical basis for integrated geriatric care.

## 2 Materials and methods

### 2.1 Participants

Older patients treated at the First People’s Hospital of Zunyi from March 2020 to March 2024 were selected. Inclusion criteria: ① age ≥60 years; ② clear consciousness, Mini-Mental State Examination (MMSE) score ≥17, and ability to complete questionnaires; ③ voluntary participation with signed informed consent. Exclusion criteria: ① severe cognitive impairment (MMSE < 17); ② severe mental illness (e.g., schizophrenia, bipolar disorder); ③ terminal physical diseases; ④ major life events in the past month; ⑤ long-term use of opioid analgesics, antidepressants, or other drugs affecting pain, sleep, and mood.

**Figure 2:**
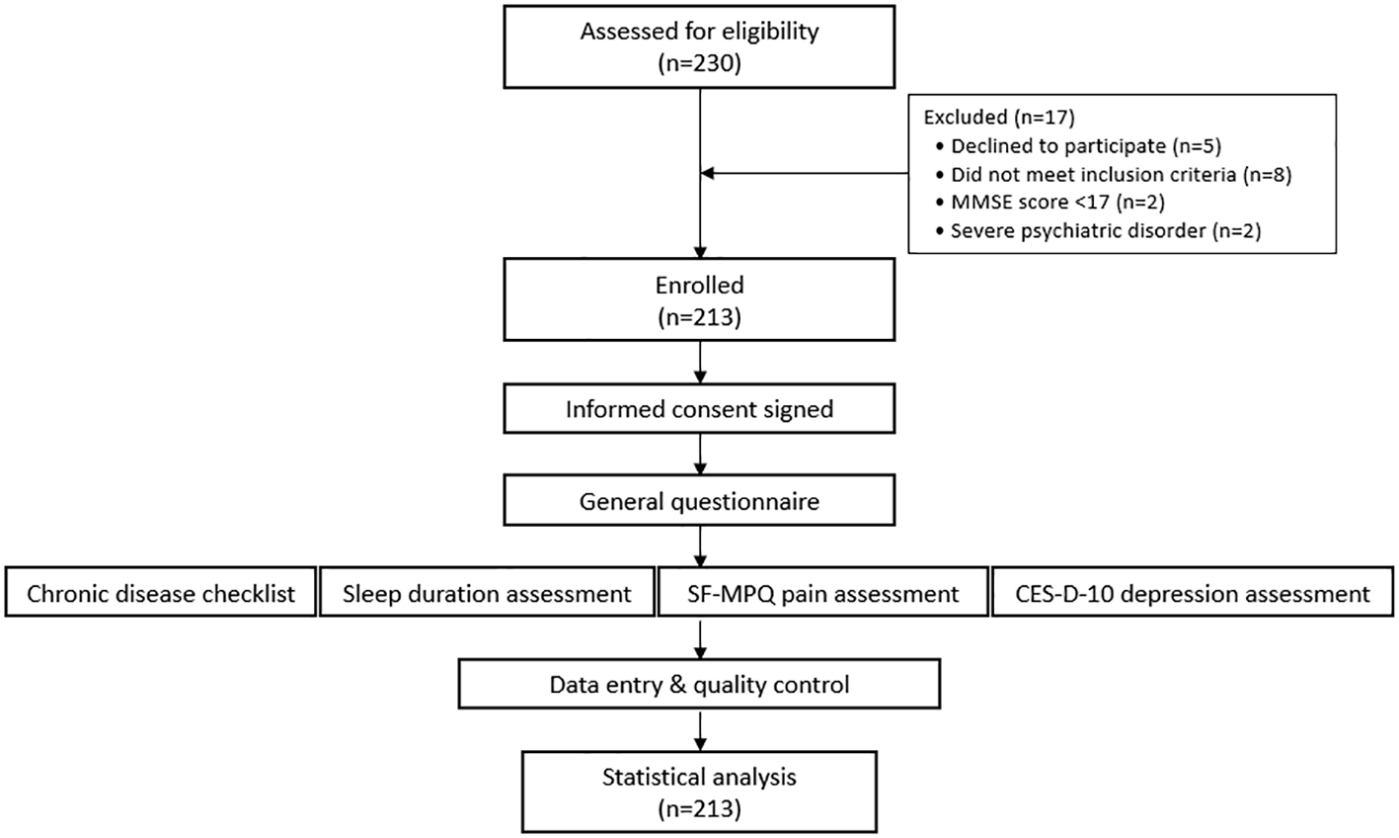
CONSORT flow diagram illustrating participant recruitment, exclusion, and study procedures.

Sample size: Based on Kendall’s principle (5–10 times the number of scale items), we required 185–370 participants. Allowing 20% invalid responses, we distributed 230 questionnaires and obtained 213 valid responses (effective rate 92.61%; **Figure 2**).

### 2.2 Survey instruments

1. General Information Questionnaire: Included age, gender, education, smoking/drinking, marital status, endowment insurance, place of residence, and sleep duration (a single item from CHARLS: “In the past month, how many hours did you actually sleep at night?”)
2. Chronic Disease Count: Based on 15 chronic diseases listed in CHARLS (hypertension, dyslipidemia, diabetes, cancer, liver disease, heart disease, stroke, kidney disease, stomach/digestive disease, emotional/mental disease, memory-related disease, arthritis/rheumatism, asthma, etc.). Participants were asked: “Have you ever been diagnosed with any of the following by a doctor?” Each disease was scored 1, and the total score was 0–15.
3. Somatic Pain: The Short-Form McGill Pain Questionnaire (SF-MPQ) was used to evaluate the somatic pain level of patients (15). The SF-MPQ consists of three parts: the first part includes 11 sensory and 4 affective descriptors, each of which requires the patient to rate the intensity on a scale of 0 (no pain), 1 (mild pain), 2 (moderate pain), and 3 (severe pain) to calculate the Pain Rating Index (PRI); the second part is the Visual Analog Scale (VAS), with scores ranging from 0 mm (no pain) to 100 mm (severe pain); the third part is the Present Pain Intensity (PPI), scored as 0 (no pain), 1 (mild discomfort), 2 (discomfort), 3 (distressing), 4 (horrible), and 5 (excruciating). The total score was the sum of PRI, VAS, and PPI scores, with higher scores indicating more severe pain.
4. Depressive Symptom Assessment: The 10-item Short-Form Center for Epidemiological Studies Depression Scale (CES-D-10) was used for measurement. The CES-D-10, widely used for screening depressive symptoms in the Chinese population, uses a 0–3 scoring system. Reverse scoring was applied to items such as “I felt hopeful about the future” and “I felt happy”. The total score ranges from 0 to 30. Based on domestic studies, a total CES-D score ≥10 was used as the cutoff to distinguish between the presence and absence of depressive symptoms, with higher scores indicating more severe depressive symptoms (16). The SF-MPQ and CES-D-10 are well-validated instruments. The Cronbach’s alpha coefficients reported in previous Chinese population studies were 0.836 for the SF-MPQ-2 total scale (17) and 0.79 for the CES-D-10 (18), respectively.

### 2.3 Outcome measures

Primary observation indicators: (1) mediating effects of somatic pain and sleep duration between chronic disease count and depressive symptoms; (2) effect values and significance of each path; (3) proportion of indirect effects. Secondary observation indicators: demographic distributions and correlations.

### 2.4 Statistical analysis

SPSS 26.0 statistical software and the PROCESS 4.1 macro program developed by Hayes were used for data analysis. Measurement data conforming to the normal distribution were expressed as mean ± standard deviation (x ± s), and t-tests or analysis of variance (ANOVA) were used for inter-group comparisons; count data were expressed as cases (%) and chi-square tests were used for inter-group comparisons. Multicollinearity was assessed by the Variance Inflation Factor (VIF); all VIFs <5, indicating no severe collinearity. No missing data were present. We tested a chain mediation model (Model 6) with chronic disease count as the independent variable (X), somatic pain as M1, sleep duration as M2, and depressive symptoms as Y. Covariates (age, gender, education, marital status, residence, smoking, drinking, endowment insurance) were included. Bootstrap with 5000 resamples generated 95% confidence intervals (CIs); CIs excluding 0 indicated significant indirect effects.

## 3 Results

### 3.1 Baseline data

Among 213 participants aged 60–84 years, 89 (41.78%) were male, 124 (58.22%) female; 31.3% urban, 68.7% rural. Mean chronic disease count = 1.97 ± 1.12; mean somatic pain score = 13.63 ± 7.37; mean sleep duration = 6.13 ± 1.72 hours; mean depression score = 9.13 ± 5.03. Depression was detected in 86 patients (40.38%; **Table 1**). Descriptive comparisons by gender showed that females had higher pain scores and shorter sleep duration than males (**Supplementary Table 1**).

**Table 1:** Baseline data of the subjects.

Baseline data	Category	n	Proportion (%)
Age (years)	60–69	135	63.38
	70–79	66	30.99
	≥80	12	5.63
Gender	Male	89	41.78
	Female	124	58.22
Education Level	Junior High School and Below	157	73.71
	Senior High School and Above	56	26.29
Marital Status	Unmarried	52	24.41
	Married	161	75.59
Place of Residence	Urban	104	48.83
	Rural	109	51.17
Smoking History	Yes	92	43.19
Drinking History	Yes	55	25.82
Endowment Insurance	Yes	58	27.23
Depression	Yes	86	40.38

### 3.2 Correlation analysis of main variables

Pearson correlations (**Table 2**) showed that chronic disease count was significantly positively correlated with somatic pain (r = 0.405, P < 0.001) and depressive symptoms (r = 0.485, P < 0.001), and negatively correlated with sleep duration (r = -0.431, P < 0.001). Somatic pain was negatively correlated with sleep duration (r = -0.431, P < 0.001) and positively correlated with depressive symptoms (r = 0.590, P < 0.001). Sleep duration was negatively correlated with depressive symptoms (r = -0.499, P < 0.001).

**Table 2:** correlation analysis of chronic disease count, somatic pain score, and depressive symptoms in older adults.

Variable	Mean ± SD	Chronic disease count	Sleep duration
Chronic Disease Count	1.97 ± 1.12	–	
Sleep Duration	6.13 ± 1.72	-0.431^***^	–
Somatic Pain	13.63 ± 7.37	0.405^***^	-0.431^***^
Depressive Symptoms	9.13 ± 5.03	0.485^***^	-0.499^***^

***P<0.001

### 3.3 Chain mediation model: regression analysis

To test the hypothesized chain mediation model, we conducted a series of regression analyses with chronic disease count as the independent variable (X), somatic pain as the first mediator (M1), sleep duration as the second mediator (M2), and depressive symptoms as the dependent variable (Y). All models were adjusted for covariates, including gender, age, education level, marital status, place of residence, smoking history, drinking history, and endowment insurance (variable coding shown in **Table 3**). Multicollinearity was assessed using variance inflation factors (VIFs); all VIFs were <5, indicating no severe collinearity. No missing data were present in the 213 valid questionnaires.

**Table 3:** Variable assignment.

Variable	Assignment
Age	60–69 years=1,70–79 years=2,≥80 years=3
Gender	Female=0, Male=1
Place of Residence	Urban=1, Rural=0
Marital Status	Married=1, Unmarried=0
Education Level	Junior High School and Below=0, Senior High School and Above=1
Smoking History	Yes=1, No=0
Drinking History	Yes=1, No=0
Endowment Insurance	Yes=1, No=0

As shown in **Table 4** (Models 1–4): Model 1: Chronic disease count had a significant positive association with depressive symptoms (β = 0.359, P < 0.001), establishing the total effect. Model 2: Chronic disease count was significantly positively associated with somatic pain (β = 0.350, P < 0.001). Model 3: Both chronic disease count (β = -0.263, P < 0.001) and somatic pain (β = -0.254, P < 0.001) were significantly negatively associated with sleep duration. Model 4: After entering somatic pain and sleep duration as mediators, the direct effect of chronic disease count on depressive symptoms remained significant but was attenuated (β = 0.164, P < 0.01). Somatic pain was significantly positively associated with depressive symptoms (β = 0.348, P < 0.001), and sleep duration was significantly negatively associated with depressive symptoms (β = -0.206, P < 0.001). The R² values for Models 1 through 4 were 0.427, 0.254, 0.335, and 0.575, respectively, indicating that the full model explained 57.5% of the variance in depressive symptoms.

**Table 4:** Regression analysis for the chain mediation model.

	Depressive symptoms	Somatic pain	Sleep duration	Depressive symptoms
	Model 1	Model 2	Model 3	Model 4
constant	0.055	0.380	-0.054	-0.109
Chronic Disease Count	0.359^***^	0.350^***^	-0.263^***^	0.164^**^
Somatic Pain			-0.254^***^	0.348^***^
Sleep Duration				-0.206^***^
Gender	-0.840^***^	-0.618^***^	0.428^**^	-0.504^***^
Education Level	0.557^***^	0.286	-0.372^*^	0.365^**^
Place of Residence	0.346^**^	0.028	-0.072	0.320^**^
Marital Status	0.185	0.087	0.015	0.153
Age	0.020	-0.154	0.103	0.103
Smoking History	-0.294^*^	-0.212	-0.298^*^	-0.271^**^
Drinking History	-0.510^***^	0.034	0.213	-0.480^***^
Endowment Insurance	0.098	0.035	-0.104	0.062
R^2^	0.427	0.254	0.335	0.575
F	16.798^***^	7.677^***^	10.154^***^	24.676^***^

*P<0.05, ^**^P<0.01, ^***^P<0.001.

### 3.4 Mediation effect analysis

**Figure 3:**
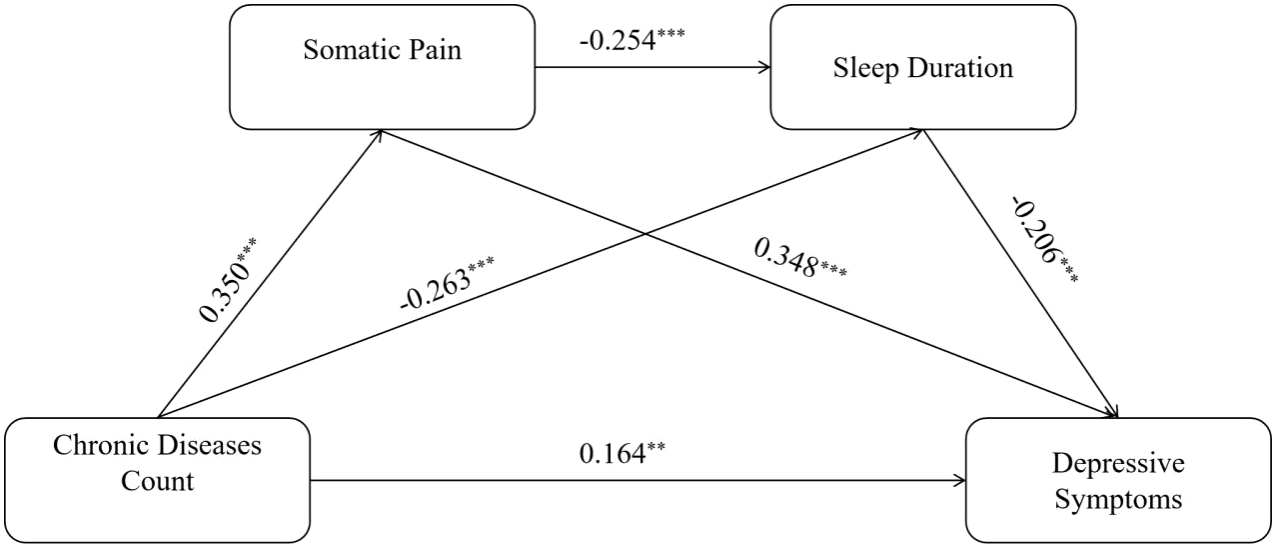
Sequential mediation model of somatic pain and sleep duration in the association between chronic disease count and depressive symptoms in older adults. Values on the arrows are unstandardized regression coefficients (β) from **Table 4**, with corresponding 95% confidence intervals shown in parentheses. Bootstrap estimates of indirect effects are presented in **Table 5**. ^*^P < 0.05, ^**^P < 0.01, ^***^P < 0.001.

To formally test the significance of indirect effects, we employed the bias-corrected nonparametric percentile bootstrap method with 5,000 resamples (PROCESS Model 6). As shown in **Table 5** and **Figure 3**: The total effect of chronic disease count on depressive symptoms was 0.359 (95% CI: 0.245–0.472). The direct effect (chronic disease count → depressive symptoms, after accounting for mediators) was 0.164 (95% CI: 0.056–0.273), accounting for 45.83% of the total effect. Total indirect effect (via somatic pain and/or sleep duration) was 0.194 (95% CI: 0.115–0.271), accounting for 54.17% of the total effect (forest plot of the indirect effects is provided in **Supplementary Figure 1**).

**Table 5:** Bootstrap analysis of indirect effects.

Path relationship	Effect value	SE	95%CI	Proportion of total effect (%)
Total Effect	0.359	0.058	0.245-0.472	100.00
Direct Effect (Chronic Disease Count → Depressive Symptoms)	0.164	0.055	0.056-0.273	45.83
Total Mediating Effect	0.194	0.040	0.115-0.271	54.17
Chronic Disease Count → Somatic Pain → Depressive Symptoms	0.122	0.033	0.057-0.187	33.90
Chronic Disease Count → Sleep Duration → Depressive Symptoms	0.054	0.019	0.021-0.096	15.14
Chronic Disease Count → Somatic Pain → Sleep Duration → Depressive Symptoms	0.018	0.008	0.006-0.037	5.13

The three specific indirect pathways were all statistically significant: Chronic Disease Count → Somatic Pain → Depressive Symptoms: effect = 0.122 (95% CI: 0.057–0.187), accounting for 33.90% of the total effect. Chronic Disease Count → Sleep Duration → Depressive Symptoms: effect = 0.054 (95% CI: 0.021–0.096), accounting for 15.14% of the total effect. Chronic Disease Count → Somatic Pain → Sleep Duration → Depressive Symptoms (chain mediation): effect = 0.018 (95% CI: 0.006–0.037), accounting for 5.13% of the total effect.

To assess the plausibility of alternative temporal sequencing, we also tested a reverse-path model (Chronic Disease Count → Sleep Duration → Somatic Pain → Depressive Symptoms) using the same covariates and estimation procedure. The proposed model yielded R² = 0.575 for depressive symptoms, compared to R² = 0.571 for the alternative model. However, as cross-sectional data cannot establish causal direction, this comparison merely indicates which pathway is more consistent with the observed data. Causal inferences are not warranted.

## 4 Discussion

This study examined the chain mediating roles of somatic pain and sleep duration in the association between chronic disease count and depressive symptoms among older hospitalized patients. Using a revised and temporally plausible model (Chronic Disease Count → Somatic Pain → Sleep Duration → Depressive Symptoms), our findings demonstrated that both somatic pain and sleep duration independently and sequentially mediated this relationship. The total indirect effect accounted for 54.17% of the total association, with the specific chain-mediated effect (Chronic Disease Count → Somatic Pain → Sleep Duration → Depressive Symptoms) being statistically significant but relatively small in magnitude (effect = 0.018, 5.13% of the total effect). Below, we discuss each pathway, integrate our findings with existing literature, address clinical implications, and critically evaluate the limitations.

### 4.1 Independent mediating effects of somatic pain and sleep duration

Consistent with previous studies (19, 20), our correlation analysis confirmed that a greater number of chronic diseases was positively associated with somatic pain and depressive symptoms and negatively associated with sleep duration. The depression detection rate of 40.38% aligns with findings from Yu et al. (21) (39.86%) but differs from other community-based reports (22, 23), likely due to variations in screening instruments (CES-D-10 vs. other scales) and sample characteristics (hospital-based vs. community-based). This high prevalence underscores the urgent need for mental health screening in older adults with chronic conditions, particularly in clinical settings where physical complaints often overshadow psychological distress.

Somatic pain as a mediator. Our first indirect pathway (chronic disease count → somatic pain → depressive symptoms) was significant, with an effect of 0.122 (33.90% of the total effect). This indicates that a substantial portion of the association between multimorbidity and depression operates through the experience of bodily pain. This finding is biologically and clinically plausible. Chronic diseases such as arthritis, heart disease, and diabetes are frequently accompanied by persistent inflammatory processes (24, 25). Elevated levels of pro-inflammatory cytokines, e.g., TNF-α, IL-1β, IFN-γ) are common in patients with multiple chronic conditions and have been shown to sensitize peripheral and central pain pathways, leading to heightened pain perception (26, 27). Beyond direct biological mechanisms, chronic illness imposes functional limitations—reduced mobility, fatigue, and social withdrawal—that can exacerbate pain through deconditioning and increased psychological distress (28, 29). Park et al. (29) and Ma et al. (30) reported that somatic pain frequently coexists with anxiety and depression, and that pain-related disability is associated with increased risk of depressive disorders. Thus, for older adults with multimorbidity, routine pain assessment should be integrated into depression screening protocols, as simply managing the underlying diseases without addressing pain may leave a key driver of depressive symptoms untreated.

Sleep duration as a mediator. The second independent pathway (chronic disease count → sleep duration → depressive symptoms) was also significant, with an effect of 0.054 (15.14% of the total effect). This confirms that shorter sleep duration is another important mechanism linking multimorbidity to depression. Several mechanisms explain this link. Many chronic diseases—particularly cardiovascular disorders, diabetes, and arthritis—are associated with nighttime symptoms such as dyspnea, nocturia, or joint pain that directly fragment sleep (31). Chronic inflammation and dysregulated cortisol secretion (HPA axis hyperactivity) are common in multimorbid patients and are associated with disrupted circadian rhythms and reduced slow-wave sleep (7, 8, 32). Additionally, the psychological burden of managing multiple conditions—medication side effects, frequent clinic visits, and health-related worry—can prolong sleep latency and reduce total sleep time (33). Our findings align with Ye and Wang (34), who reported that multimorbidity was associated with poor sleep in Chinese older adults. However, our study extends this by positioning sleep duration as a specific mediator of the disease–depression relationship. Importantly, emerging evidence suggests that the association between sleep and depression may be non-linear, with both short (<6h) and long (>9 h) sleep durations conferring increased risk (31, 35). Due to our relatively small sample and limited number of participants with very long sleep duration, we analyzed sleep linearly; thus, our mediation estimate may represent a conservative approximation. Future studies with larger samples and multidimensional sleep assessments (e.g., PSQI, actigraphy) should examine potential non-linear or threshold effects.

### 4.2 Chain mediating effect: statistical significance but limited clinical utility

The sequential pathway (chronic disease count → somatic pain → sleep duration → depressive symptoms) yielded a statistically significant effect of 0.018 (95% CI: 0.006–0.037), accounting for 5.13% of the total effect. While this confirms the hypothesized cascade, the effect size is extremely small in absolute terms. On the CES-D-10 scale (range 0–30), an effect of 0.018 corresponds to a clinically negligible change—far below the minimal clinically important difference (MCID) typically defined for depression screening tools. This means that, although the sequential pathway is statistically detectable, its real-world clinical utility is very limited.

This finding carries important implications for intervention design. First, it suggests that the indirect effect of multimorbidity on depression is not driven predominantly by the long chain but rather by the independent, direct effects of pain and sleep deprivation. Second, from an intervention standpoint, targeting pain and sleep directly—rather than attempting to modify chronic disease count (a relatively stable, long-term characteristic)—may be a more efficient and clinically meaningful strategy for reducing depression risk. For instance, cognitive-behavioral therapy for pain and insomnia, physical activity programs, and sleep hygiene education could yield larger and more immediate mental health benefits than disease-focused management alone.

Nevertheless, the statistical significance of the chain effect should not be dismissed entirely. It provides confirmatory evidence for the biopsychosocial model, demonstrating that biological vulnerability (multimorbidity) may operate through physical (pain) and behavioral/physiological (sleep) mechanisms to affect psychological well-being. This theoretical validation is valuable for guiding future longitudinal research and for understanding the complex interconnections among physical and mental health in later life.

### 4.3 Comparison with previous studies and theoretical integration

Our model aligns with the work of Zhang et al. (20), who positioned chronic disease count as an upstream variable with somatic pain and depression as parallel mediators. However, our study extends Zhang’s framework by adding sleep duration as a second sequential mediator. While Zhang et al. demonstrated that pain and depression independently mediate the disease-sleep relationship, we show that pain and sleep form a cascading pathway—disease is associated with pain, which in turn is associated with sleep disruption, and both contribute to depression. This provides a more nuanced understanding of how multimorbidity impacts mental health and offers multiple intervention targets (pain management and sleep regulation) along the same causal chain.

Our findings also complement those of Luo et al. (19) and Read et al. (36), who emphasized that pain and depression are tightly intertwined in multimorbid patients, and that healthcare utilization is significantly higher in those with co-occurring physical and mental symptoms. By delineating the sequential pathway, we provide a clearer rationale for integrated care models that address pain and sleep as core components of geriatric depression prevention.

To further assess the plausibility of our hypothesized sequencing, we compared our proposed model with an alternative reverse-path model. The comparison showed slightly better fit for our proposed model. However, consistent with the cross-sectional nature of our data, this result does not confirm temporal precedence; it merely indicates which pathway is more consistent with the observed associations. We therefore interpret our findings with caution, recognizing that only longitudinal designs can definitively address questions of causal ordering.

Taken together, our findings fit coherently within the biopsychosocial model of health and illness. Chronic multimorbidity represents a biological stressor that imposes systemic inflammation and functional impairment. This biological burden manifests as somatic pain—a physical and psychological experience that is not only associated with direct distress but also disrupts sleep duration through neuroendocrine and behavioral mechanisms. Poor sleep, in turn, impairs emotional regulation, reduces coping resources, and exacerbates negative cognitive biases, ultimately contributing to depressive symptoms. This cascade illustrates how physical health, behavior, and mental health are dynamically interconnected in later life and underscores the need for multidisciplinary, holistic geriatric care that transcends traditional specialty boundaries.

### 4.4 Clinical implications

Our results offer several actionable clinical insights for geriatric practice: First of all, routine pain and sleep screening should be integrated into depression prevention programs for older adults with multimorbidity, as these are modifiable factors that independently and directly associate with depressive symptoms. Then, interventions targeting pain (e.g., multidisciplinary pain management, physical therapy, cognitive-behavioral therapy for pain) and sleep (e.g., sleep hygiene, cognitive-behavioral therapy for insomnia, chronotherapy) may yield more immediate and clinically significant mental health benefits than interventions focused solely on disease count modification. The very small chain-mediated effect suggests that long-chain interventions (i.e., attempting to modify depression by sequentially targeting diseases, then pain, then sleep) are unlikely to be clinically efficient. Instead, direct, symptom-focused approaches are recommended. Moreover, healthcare systems should consider integrated care pathways that combine chronic disease management, pain services, and sleep medicine within a single geriatric mental health framework to reduce fragmentation and improve outcomes.

Sex-sensitive considerations. Although we could not perform formal sex-stratified mediation analyses due to sample size constraints, our descriptive comparisons (**Supplementary Table 1**) revealed notable sex differences: female participants reported significantly higher somatic pain scores (14.71 vs. 12.13, P = 0.011), shorter sleep duration (5.93 vs. 6.42 hours, P = 0.036), and higher depressive symptom scores (9.98 vs. 7.82, P = 0.002) than males. These patterns are consistent with well-established epidemiological findings that women are at greater risk for chronic pain, sleep disturbances, and depression across the lifespan (37). Biologically, sex hormones (particularly estrogen fluctuations) modulate pain perception, inflammatory responses, and circadian rhythms (37). Psychologically, women may be more likely to report pain and emotional distress, and may also bear greater caregiving and household responsibilities that exacerbate sleep disruption and psychological burden (29, 30). These descriptive findings suggest that the mediating pathways we identified may operate with greater magnitude in women, and clinicians should be particularly attentive to pain and sleep complaints in older female patients. Future studies with adequately powered samples should formally test whether sex moderates the proposed mediation pathways, which could inform tailored intervention strategies.

### 4.5 Limitations

Several limitations should be acknowledged. First, the single-center, hospital-based sample may limit generalizability to community-dwelling populations. Second, the cross-sectional design precludes causal inferences; although our model is theoretically plausible and showed slightly better fit (R² = 0.575 vs. 0.571) than an alternative reverse-path model, this fit comparison does not constitute evidence of causality. The chain mediation hypothesis was theoretically derived, and only longitudinal studies can establish temporal ordering. Third, all variables were self-reported, subject to recall and reporting bias. Fourth, sleep duration was assessed using a single item, which does not capture sleep quality, efficiency, or daytime dysfunction. Fifth, we could not report Cronbach’s α for the SF-MPQ and CES-D-10 specific to our sample because individual item data were not retained—a regrettable data management limitation, although both scales are well validated in Chinese populations (17, 18). Sixth, chronic disease burden was measured as a simple count, not accounting for severity, duration, or specific disease combinations. Seventh, several potential confounders (social support, physical activity, cognitive function) were not included. Eighth, the sample size was insufficient for sex-stratified mediation analyses, although descriptive data showed sex differences that warrant future investigation. Ninth, the small effect size of the chain-mediated pathway limits its clinical relevance. Despite these limitations, our study provides useful empirical evidence and a framework for future research.

## 5 Conclusions

In conclusion, somatic pain and sleep duration independently and sequentially mediate the association between chronic disease count and depressive symptoms among older hospitalized patients. The findings support a biopsychosocial cascade in which multimorbidity is associated with increased pain, which in turn is associated with shortened sleep, and both pain and sleep disruption are associated with greater depressive symptoms. However, the chain-mediated effect was extremely small (0.018, 5.13% of the total effect), suggesting that direct interventions targeting pain and sleep—rather than long-chain approaches—may offer more clinically meaningful benefits for reducing depression risk in older adults. Given the cross-sectional design, causal directions cannot be inferred. Future prospective studies with objective measures, larger samples, and comprehensive psychosocial assessments are urgently needed to verify these associations, explore non-linear relationships, and examine sex-specific pathways.

## Data Availability

The original contributions presented in the study are included in the article/**Supplementary Material**. Further inquiries can be directed to the corresponding author.

## Supplementary material

Supplementary Table 1 Gender-stratified descriptive statistics of major variables. Data are presented as mean ± standard deviation. P-values were obtained from independent-samples t-tests (two-tailed). SF-MPQ, Short-Form McGill Pain Questionnaire; CES-D-10, 10-item Center for Epidemiological Studies Depression Scale.

Supplementary Figure 1 Forest plot of indirect effects from the chain mediation model. Squares indicate point estimates; horizontal lines represent 95% confidence intervals. CIs excluding zero indicate statistical significance. All estimates are unstandardized coefficients based on 5,000 bootstrap resamples.
