# Supplementary material for: Chain mediating effect of somatic pain and sleep duration between chronic disease count and depressive symptoms in older adults
Source: Front Psychiatry. 2026 Jul 22;17:1821807. doi: 10.3389/fpsyt.2026.1821807 (PMC13438298; doi:10.3389/fpsyt.2026.1821807)
Supplement: Supplementary file 1 [file DataSheet1.docx]

**Chain Mediating Effect of Somatic Pain and Sleep Duration Between Chronic Disease Count and Depressive Symptoms in Older Adults**

**Lan Ma**

Department of Pain, The First People's Hospital of Zunyi, 563000, Guizhou, China

Email: 18984215468@163.com

**Keywords:** Chronic Diseases; Sleep Duration; Somatic Pain; Depressive Symptoms; older adults; Chain Mediating Effect

Supplementary Table S1. Gender-stratified descriptive statistics of major variables

| Variable | Male (n = 89) | Female (n = 124) | t | p |
| --- | --- | --- | --- | --- |
| Chronic disease count | 1.92 ± 1.09 | 2.01 ± 1.14 | −0.58 | 0.562 |
| Sleep duration (hours) | 6.42 ± 1.61 | 5.93 ± 1.76 | 2.11 | 0.036 |
| Somatic pain score (SF-MPQ total) | 12.13 ± 6.85 | 14.71 ± 7.52 | −2.58 | 0.011 |
| Depressive symptoms (CES-D-10) | 7.82 ± 4.66 | 9.98 ± 5.08 | −3.18 | 0.002 |

Note: Data are presented as mean ± standard deviation. P-values were obtained from independent-samples t-tests (two-tailed).

Abbreviations: SF-MPQ, Short-Form McGill Pain Questionnaire; CES-D-10, 10-item Center for Epidemiological Studies Depression Scale.

Figure S1 Forest plot of indirect effects from the chain mediation model. Squares indicate point estimates; horizontal lines represent 95% confidence intervals. CIs excluding zero indicate statistical significance. All estimates are unstandardized coefficients based on 5,000 bootstrap resamples.
